# Diet, Gut Microbiome and Epigenetics: Emerging Links with Inflammatory Bowel Diseases and Prospects for Management and Prevention

**DOI:** 10.3390/nu9090962

**Published:** 2017-08-30

**Authors:** Krasimira Aleksandrova, Beatriz Romero-Mosquera, Vicent Hernandez

**Affiliations:** 1 Nutrition, Immunity and Metabolism Start-up Lab, Department of Epidemiology, German Institute of Human Nutrition Potsdam-Rehbrücke, Arthur-Scheunert Allee 114-116, 14558 Nuthetal, Germany; 2 Department of Gastroenterology, Instituto Investigación Sanitaria Galicia Sur, Estrutura Organizativa de Xestión Integrada de Vigo, 36312 Vigo, Spain; beatriz.romero.mosquera@sergas.es (B.R.-M.); vicenthernandez@yahoo.es (V.H.)

**Keywords:** diet, gut microbiota, epigenetics, inflammatory bowel diseases

## Abstract

Inflammatory bowel diseases (IBD) represent a growing public health concern due to increasing incidence worldwide. The current notion on the pathogenesis of IBD is that genetically susceptible individuals develop intolerance to dysregulated gut microflora (dysbiosis) and chronic inflammation develops as a result of environmental triggers. Among the environmental factors associated with IBD, diet plays an important role in modulating the gut microbiome, influencing epigenetic changes, and, therefore, could be applied as a therapeutic tool to improve the disease course. Nevertheless, the current dietary recommendations for disease prevention and management are scarce and have weak evidence. This review summarises the current knowledge on the complex interactions between diet, microbiome and epigenetics in IBD. Whereas an overabundance of calories and some macronutrients increase gut inflammation, several micronutrients have the potential to modulate it. Immunonutrition has emerged as a new concept putting forward the importance of vitamins such as vitamins A, C, E, and D, folic acid, beta carotene and trace elements such as zinc, selenium, manganese and iron. However, when assessed in clinical trials, specific micronutrients exerted a limited benefit. Beyond nutrients, an anti-inflammatory dietary pattern as a complex intervention approach has become popular in recent years. Hence, exclusive enteral nutrition in paediatric Crohn’s disease is the only nutritional intervention currently recommended as a first-line therapy. Other nutritional interventions or specific diets including the Specific Carbohydrate Diet (SCD), the low fermentable oligosaccharides, disaccharides, monosaccharides, and polyol (FODMAP) diet and, most recently, the Mediterranean diet have shown strong anti-inflammatory properties and show promise for improving disease symptoms. More work is required to evaluate the role of individual food compounds and complex nutritional interventions with the potential to decrease inflammation as a means of prevention and management of IBD.

## 1. Introduction

**Figure 1:**
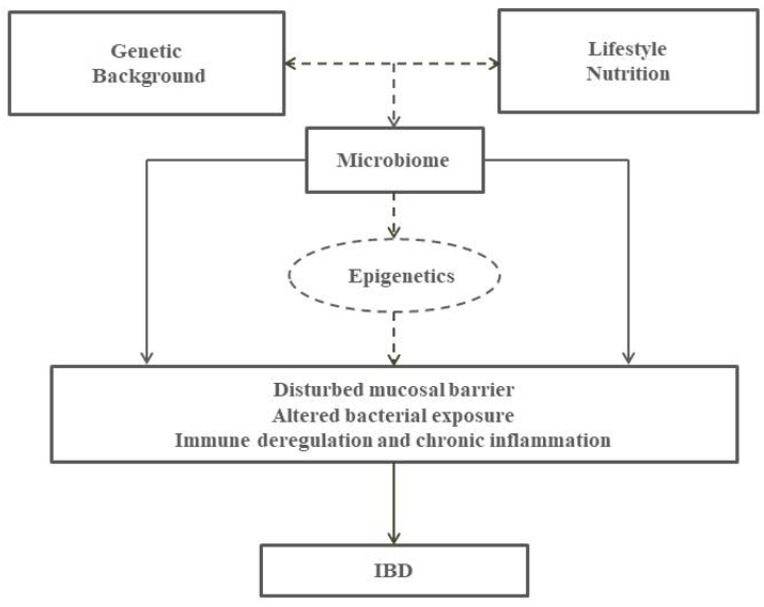
Proposed patho-physilogical mechanisms for inflammatory bowel disease (IBD). Complex interaction between genetic and lifestyle factors and the putative role of epigenetics on the interplay between microbiota, the immune system and the mucosal barrier.

The inflammatory bowel diseases (IBD)—Crohn’s disease (CD) and ulcerative colitis (UC)—are two diseases characterised by chronic relapsing inflammation of the gastrointestinal tract. They represent an increasing public health concern and an aetiological enigma due to unknown causal factors. Despite suggested differences in pathology, both diseases are believed to share a common aetiology. The strongest IBD risk factor identified to date is a family history of IBD [1]. The current notion on the pathogenesis of IBD is that genetically susceptible individuals develop intolerance to dysregulated gut microflora (dysbiosis) and chronic inflammation develops as a result of environmental triggers [2]. Current research in the field of IBD largely focusses on establishing the role of causal variants on gene expression and various pathological pathways have been already uncovered [3]. However, genetic risk loci identified to date only explain a small part of the genetic variance in disease risk and more factors need to be taken into account for understanding the multifactorial pathology of IBD [4]. Impaired immune response in genetically susceptible individuals as the result of a complex interaction between impaired intestinal barrier function and dysfunctional microbe–host interactions has therefore been suggested as a major unifying aetiological background [5]. While the identification of IBD environmental risk factors remains a subject of intensive research, diet remains one of the best candidates. Diet participates in the regulation of intestinal inflammation, either directly or indirectly by modifying the gut microbiota [6,7]. A greater understanding of the contribution of dietary factors to dysbiosis is therefore critical as a healthy microbiome is pivotal in preventing the development of IBD and its complications [8]. Most recently, the fast-evolving field of epigenetics has offered new explanations on the mechanisms by which environmental changes induce pathological gene expression and determine cell phenotype and function in IBD [9]. Collectively, the pathogenic mechanisms for IBD could be largely imposed by gene–environment interactions switching on a cascade of induced effects on the microbiota, the immune system, and the mucosal barrier (Figure 1).

Here, we review the recent developments in understanding the role of the gut microbiome, epigenetics, and dietary factors in IBD that outline directions for disease management and prevention.

## 2. Epidemiology of IBD and Environmental Exposures

IBDs occur worldwide with differences in epidemiology, exposures to risk factors and phenotypes between regions. The prevalence of IBD is higher in industrialised countries (e.g., Western Europe, United States of America, Canada, Australia and New Zealand) than in developing countries (in Asia, the Middle East, South America and Africa) [10]. The incidence of IBD steadily increased in industrialised countries during the 20th century and, although some studies indicated that a plateau was reached in some regions during the 21st century [10], recent reports suggest that it could be still increasing in these countries [11,12,13]. In developing countries, traditionally considered low-incidence areas, increasing incidence has been described since the beginning of the 21st century [8,9,10,14]. These observed increases in incidence rates could be partially accounted for by pragmatic reasons such as increased media coverage and health awareness in both developed and developing countries, improved access to medical technology and health care providers, and the development of sophisticated disease surveillance systems [15,16]. However, the parallel of higher incidence rates with the Westernisation of affected societies points to the potential important role of the environment [15,16]. In that regard, environmental factors (also known as “exposome”) have been thoroughly studied and many of the factors related to Westernisation have been linked to the risk of IBD development. The list of putative factors so far includes environmental pollution, medication, psychological stress, infections and lifestyle. Certain differences in environmental risk for CD and UC have been reported. For example, cigarette smoking has been shown to increase the risk of developing CD, while smoking is less common in people at risk for UC [17]. Studies of migrant populations moving from regions of low to high IBD incidence point to early life as a key time for environmental triggers [18]. In these populations, the second generation, i.e., those born in high-incidence regions, have been shown to have higher incidence rates compared to their parents. Early life environmental exposures, such as breast feeding, have been also implicated in IBD risk [18]. However, an important obstacle to identifying the role of environmental factors in IBD is a lack of methodological standardisation among studies [19].

## 3. Intestinal Microbiome in the Pathogenesis of IBD

The gut microbiome interacts with the host in a symbiotic way and exerts a variety of beneficial effects including digestion of substrates and nutrient production; development, maturation and regulation of the immune system (both the local and the systemic response); and prevention of the growth of harmful microorganisms [20]. The human microbiota has been classified into clusters of well-balanced, defined microbial community compositions, known as “enterotypes” [21]. While the enterotypes are stable, environmental factors (i.e., diet) can influence microbiome composition without affecting the enterotype entity [22,23]. IBD is associated with alterations in the composition of the intestinal microbiota, characterised by decreased diversity, reduced proportions of Firmicutes, and increased proportions of Proteobacteria and Actinobacteria [6]. Some of the bacterial species with pro-inflammatory actions are enriched in patients with IBD (Escherichia, Fusobacterium), while anti-inflammatory species (Faecalibacterium, Roseburia) are largely reduced in IBD [6]. Patients with active IBD have different microbial composition as compared to patients in remission. For example, patients with active IBD have been shown to have lower abundance of *Clostridium coccoides*, *Clostridium leptum*, *Faecalibacterium prausnitzii* and Bifidobacterium [24]. Prospective studies investigating microbiome changes during disease course have been scarce. A Dutch study based on 10 CD and nine UC patients reported on patient-specific shifts in microbial composition, but could not demonstrate general changes in microbial composition or diversity [25]. A Spanish study followed up with 18 UC patients over the course of one year; in those who remained in remission *Faecalibacterium prausnitzii* increased steadily, while in those who relapsed it remained low [26].

Several factors could potentially influence the microbiome’s composition. The intestinal colonisation begins immediately after birth, and beyond genetic predisposition is influenced very early in life by the route of delivering, the infant diet (breastfeeding or formula) and by hygiene. Apart from environmental factors such as drugs, stress and toxins (i.e., tobacco), diet was suggested to play a decisive role in modulating the microbiome composition [6,20,27]. Dietary composition was shown to affect the microbiota balance; therefore, it is conceivable that altering the diet can impact the inflammatory response [28]. For example, diets rich in saturated fats are shown to induce damage of the intestinal epithelial cell layer, leading to a loss of barrier function in animal studies [29,30]. In contrast, diets high in fibre predispose short-chain fatty acid production by the microbiota and lead to improved energy expenditure [31]. Thus, a balanced low-fat and high-fibre diet may be important in preventing dysbiosis and preserving the immune system [32]. Targeting microbiota through nutritional interventions could represent a promising therapeutic approach. So far, several nutritional interventions have been evaluated such as dietary supplementation with prebiotics and contrabiotics, phosphatidylcholine and the use of genetically modified bacteria [33]. Among these, therapies such as prebiotics and probiotics that selectively manipulate the intestinal microbiota have been evaluated as an attractive therapeutic option with few side effects [34]. Prebiotics represent non-digestible carbohydrates that promote the growth of beneficial bacteria in the gut. They may increase the production of short-chain fatty acids and the modulation of cytokine production within the gut mucosa [35]. Diets deficient or low in fibre were shown to exacerbate colitis development, while a very high intake of dietary fibre or short-chain fatty acids may protect against colitis [36]. Despite evidence from animal studies that suggested the beneficial actions of prebiotics in IBD [32,35], evidence from human studies is insufficient. A recent review summarised existing evidence from human randomised trials on the effect of prebiotics in UC [37]. Overall, 17 studies reported inconsistent findings for the effect of five prebiotic groups, including fructo-oligosaccharides, barley foodstuff, galacto-oligosaccharides, lactulose and resveratrol, in modulating ulcerative colitis.

Probiotics contain living bacteria that are suggested to exert positive health effects in the human intestine, modulating mucosal permeability and strengthening the immune system keeping away pathogens from intestinal mucosa surface. In particular, animal research has suggested that Lactobacillus and Bifidobacteria produce harmful substances for Gram-positive and Gram-negative bacteria and compete with pathogenic bacteria [32,38]. Further, human studies suggested that *Clostridium coccodies* and *C. leptum* exert protective effects against IBD [32]. The multispecies products VSL#3 and *E. coli* Nissle have been revealed as particularly effective in maintaining remission in UC [31]. Probiotic yogurt intake was associated with significant anti-inflammatory effects that paralleled the expansion of peripheral pool of putative T(reg) cells in IBD patients and had few effects in controls [39]. Recently, a meta-analysis based on 22 randomised control trials compared effects of administering probiotics with 5-aminosalicylates (5-ASAs) or placebo in relation to IBD [40]. Overall the meta-analysis suggested that probiotics, and VSL#3 in particular, may be as effective as 5-ASAs in preventing a relapse of quiescent UC. However, the overall efficacy of probiotics in IBD should be confirmed by further research. In addition, long-term benefits from probiotics may be limited without an overall modification of the person’s diet. Thus, single pre-/probiotic administration may not prove useful outside of the context of switching to an overall healthy diet plan. Probiotics and other commercial interventions such as tea or berry extracts would be unlikely to counteract an unhealthy diet and, used alone, may, analogous to other medicinal products such as antioxidant supplements, fail to improve primary or secondary disease prevention [41]. More effort should be put into evaluating complex lifestyle and nutritional approaches for modulating the gut microbiome. Much work remains before the understanding of the effects of dysbiosis in humans reaches that of mice; however, while definitive statements may be lacking, the preponderance of current evidence strongly suggests that the gut microbiome is a major contributor to human health and disease [32].

## 4. Epigenetics and IBD

Research in recent years has contributed to an improved understanding of the role of epigenetic modifications—i.e., non-coding RNAs and DNA methylation—in defining the molecular basis of IBD [42,43]. Such research has been largely driven by observations that genetics alone cannot explain the onset of IBD. Thus, a meta-analysis of GWAS studies estimated that susceptibility loci for UC explained only 16% of UC heritability [44]. In this regard, gene–environment interactions have been suggested to play an important role in IBD pathogenesis and this is where epigenetics could offer new insights beyond genetic research [42,45]. Epigenetic factors were therefore suggested to mediate interactions between the environment and the genome, thereby providing new insights into the pathogenesis of IBD [46]. Earlier studies reported on the differential expression of specific microRNAs in the colonic mucosa samples of IBD patients compared to the mucosa of control patients [43]. miRNAs identified in peripheral blood were additionally suggested as new biomarkers of disease development [47]. Recent data have implicated miRNAs in immune response to bacteria invasion and the differential regulation of cytokines [48]. miRNA dysregulation, especially in Th17 cells, has been implicated in IBD. miRNAs have also been shown to regulate intestinal barrier integrity in UC. As previously reviewed, an increased expression of miR-21 is among the most consistently replicated novel therapeutic targets [47,48]. More recently, DNA methylation signatures for UC and CD have been also described. However, whether changes in DNA methylation systematically correlate with gene expression is not clear [49]. In addition, it remains challenging to identify aetiologically significant epigenetic alterations since epigenetic modification of DNA may differ between tissues, time of development within the same tissue, and environmental influences.

Initial evidence arising from epigenetic research is sometimes hard to prove in clinical practice. An example is the identified role of cytokines and subsequent development of biological drugs that fail to prove an important role in disease control. Thus, dysregulation of cytokine genes and increased mRNA levels of cytokines, including interleukin1-beta, interleukin-18 and tumour necrosis factor-alpha (TNFα), were reported in IBD patients compared with controls in the late 1990s [50,51,52,53]. This has led to introducing biologically based therapies (i.e., anti-TNFα) in IBD patients. However, achieving adequate response levels still remains elusive as stand-alone anti-TNFα therapies have not proven completely useful at predicting disease progression and drug response [54]. Recently, animal models suggested that the lack of response could be related to differences in the gut microbiome before and after disease initiation. Thus, alternative strategies are needed to account for the interplay between immunity, epigenetics and dietary factors.

Diet is known to influence epigenetic changes associated with disease and to modify gene expression patterns in a state of disturbed immunity [45]. Poor dietary choices are encoded into the human gut and genetic make-up, and could be transferred to the offspring [55]. A number of nutrients have been shown to modulate immune responses and may potentially counteract inflammatory processes [56]. Recent research suggested secondary plant metabolites, such as polyphenols, may modulate gene expression, chromatin remodelling and DNA methylation [57]. Polyphenols in green tea or soybean such as epigallocatechin-3-gallate or genistein have been demonstrated to inhibit DNA methyltransferases activity. Epigenetic effects have also been shown for other dietary components such as curcumin [58]. Nutrition provides substrates necessary for DNA methylation and can regulate the activity of the enzymes involved in the one-carbon cycle. Thus, precursors of S-adenosylmethionine, such as methionine, folate, choline, betaine and vitamins B2, B6 and B12, have been suggested to influence DNA methylation patterns [59].

Immune cells are rapidly dividing and have increased sensitivity to impaired DNA replication. Dietary factors act differently to modulate immune response, but all appear to have the potential to modulate inflammation [60]. Furthermore, active immunisation against the outer membrane protein of bacteria present in the gut was recently shown to enhance local and systemic immune control via apoE-mediated immune-modulation [61]. Immunonutrition was therefore suggested as a less invasive alternative to immunotherapy in protection against chronic inflammation predisposing IBD [60].

Gut microbiota may alter host histone acetylation and methylation in human colon tissues [62]. Fermentation end products, especially short-chain fatty acids such as acetate, butyrate and propionate, which are mostly produced by microbial fermentation of fibre, may be particularly important for the epigenetic regulation of inflammatory reactions [62]. A diet poor in fibre leads to a suppression in microbiota-driven short-chain fatty acid production and disturbed chromatin effects [62]. Of note is the finding previously mentioned, that butyrate-producing bacteria (Faecalibacterium) and SCFA-producing bacteria (Roseburia) are decreased in IBD [6]. However, the therapeutic value of butyrate-producing bacteria as pharmabiotics in humans has been questioned because of the difficulty of growing them in vitro [63]. The current state of research does not allow for definitive statements on which exact changes in the diet affect epigenetics via the microbiota. Nevertheless, accumulating lines of research reveal certain microbes that communicate with their hosts by sending out metabolites influencing gene transcription in the colon and potentially driving disease development.

## 5. Diet, Immunity and IBD

Western diet is characterised by overconsumption of refined sugars, salt, and saturated fat and low consumption of dietary fibre, as well as overall low food variability. New features of human nutrition in modern society include artificial sweeteners, gluten, and genetically modified foods. Western societies seem to have dealt with micro- and macronutrient deficiencies; however, overabundance of calories and macronutrients poses new challenges of increased inflammation, infection susceptibility, and increased risk for auto-inflammatory diseases such as IBD [64,65]. Several micronutrients are especially important for immunonutrition, among which vitamins such as vitamins A, C, D and E, folic acid, beta carotene and trace elements such as zinc, selenium, manganese and iron have garnered much research interest. Deficiencies in zinc and vitamins A and D may reduce the natural killer cell function, whereas supplemental zinc or vitamin C may enhance their activity [66,67]. Vitamin D has been shown to play a role in intestinal defence by suppressing the microbial invasion into the epithelium. Vitamin D deficiency has been identified in 82% of IBD patients, compared to the 31% national average, and has been linked to defective epithelial processes. Therapy targeting vitamin D3 signalling was suggested for treatment of inflammatory diseases, affecting both innate and adaptive immune functions. The overall impact of vitamins on IBD is still not well understood. So far, only two randomised clinical trials were conducted to evaluate the effect of vitamin D supplementation on IBD outcomes. In a Danish study, 94 patients were randomised to receive oral vitamin D3 or a placebo; patients receiving vitamin D3 had a non-significant reduced risk of relapse [68]. A more recent Iranian study conducted among 108 IBD patients reported that oral supplementation with vitamin D3 reduced serum TNF-alpha levels, though not substantially [69]. More studies with larger samples would be beneficial to assess effects of vitamin supplementation in IBD. Trace elements represent another important avenue for research in the prevention and control of inflammatory diseases. Zinc is involved in the control of DNA replication and transcription and controls signal transduction during T-cell activation [70]. Selenium deficiency decreases antibody production, while selenium supplementation enhances T-cell responses and increases antibody synthesis. It is also known to exert antioxidative effects and protect against the deteriorating effects of reactive oxygen species [71]. Iron deficiency leads to defective T-cell proliferative response and impaired cytokine production by lymphocytes. It should be noted that iron supports pathogen development and iron supplementation can also result in increased susceptibility to infections [72]. Of note, dietary iron has also been shown to enhance IBD and carcinogenesis by augmenting oxidative and nitrosative stress. In an experimental animal study, an iron-enriched diet significantly increased colorectal tumour incidence as compared with the control diet [73]. Despite the fact that micronutrient deficiency may theoretically influence the immune system and predispose one to the onset and development of IBD, more research is needed to understand optimal micronutrient levels and specific therapeutic implications [28]. Beyond micronutrients, specific food compounds such as green tea [74,75,76] or Echinacea [77,78,79] have also been suggested to reduce or enhance immune stimulation and play a role in IBD prevention.

## 6. Dietary Patterns in IBD Management and Prevention

Despite years of research, the role of diet in the prevention and control of IBD is not well understood [80,81,82]. Overall, no concerted effort has been made to provide nutritional guidelines for IBD patients and the existing guidelines largely follow the principle “If it hurts, don’t do it”. Dietary recommendations include patient advice to self-monitor and avoid foods that may worsen symptoms, eating smaller meals at more frequent intervals, drinking adequate fluids, avoiding caffeine and alcohol, taking vitamin/mineral supplementation, eliminating dairy if lactose intolerant, limiting excess fat, reducing carbohydrates and reducing high-fibre foods during flares. Mixed advice exists regarding pre-/probiotics. Recommendations are different across regions/countries. For example, enteral nutrition is recommended for Crohn’s disease patients in Japan, which differs from practices in the USA [82]. A potential reason for the lack of solid dietary recommendations is the scarcity of studies evaluating the role of diet in IBD [83]. So far, only one study has assessed nutritional factors and their influence on disease outcome in newly diagnosed IBD patients [84]. In this inception cohort study, high intake of caffeine was associated with an increased risk of surgery, severe disease course and higher treatment step in CD patients; in UC patients, daily fast food intake was associated with an increased risk of surgery and high intake of caffeine was associated with a higher risk of extra-intestinal manifestations and lower treatment step.

In an attempt to fill this gap, in recent years more effort has gone into evaluating specific diets for the management of IBD, such as the Specific Carbohydrate Diet (SCD) and the low fermentable oligosaccharides, disaccharides, monosaccharides, and polyol (FODMAP) diet. Exclusive enteral nutrition is recommended as a first-line therapy to induce remission in children with active luminal CD [85]. In adults, long-term diet interventions such as total parenteral nutrition or the elemental diet [86] have also shown promise; however, their administration is more complicated and does not allow patients to lead a normal life. The SCD is a dietary regime that aims to induce and maintain drug-free remission in patients with IBD, initially developed by gastroenterologist Sidney Haas in 1951 and later popularised by biochemist Elaine Gottschall in the book *Breaking the Vicious Cycle: Intestinal Health Through Diet* [87]. The SCD diet is based on the hypothesis that patients with IBD and other intestinal diseases present a dysfunction of the disaccharidases, which are necessary to digest and absorb disaccharides and amylopectin; therefore, higher amounts of disaccharides would enter into the colon, which may lead to bacterial overgrowth and bowel injury with increased intestinal permeability. The diet allows carbohydrate foods consisting of monosaccharides only (fruit and honey) and excludes disaccharides and most polysaccharides; therefore it includes vegetables with a high amylose-to-amylopectin ratio, fruits, nuts, solid proteins and fats [87]. So far, several case-series studies have suggested an important potential of the SCD diet in the control and remission maintenance in IBD [65,88,89,90]. The low FODMAP diet gained much attention in research as means for IBD treatment. A recent meta-analysis including two randomised control trials and four before–after studies with a total of 319 patients (96% in remission) reported overall improvement in gastrointestinal symptoms such as diarrhoea response abdominal bloating fatigue and nausea [91]. Recently, plant-based dietary patterns were suggested as valid means of long-term inflammation control [92]. In particular, the Mediterranean diet was suggested to exert strong immunomodulatory effects and even showing a potential to modulate epigenetic mechanisms. Recent data from the Predimed study, a randomised, controlled, parallel trial in high cardiovascular risk volunteers, revealed that over five years of intervention the Mediterranean diet was associated with the methylation of genes related to inflammation and exerted high regulatory effects [93]. Further intervention trials utilising transcriptomics analyses revealed the potential of Mediterranean to modulate gene expression and to normalise microbiota in IBD patients [94]. Similarly, semi-vegetarian diet (SVD) was shown to exert preventive effects against IBD relapse in patients who have achieved remission in a prospective, single-centre, two-year clinical trial [95]. In summary, the current evidence on specific diets in the IBD course remains to be confirmed and updated by further well-designed and long-term follow-up studies, in which the complex interactions between nutrients and microbiota should be taken into account.

## 7. Ongoing Research

Several research projects have been recently launched or are being launched that aim to assess diet and the microbiome as related to IBD. These include the Food and Resulting Microbial Metabolites (FARMM) study by the Crohn’s and Colitis Foundation of America (CCFA) [96], the Study on the Genetic, Environmental and Microbial Interactions that Cause IBD (GEM Project) by the Crohn’s and Colitis Canada [97], the Prognostic effect of Environmental factors in Crohn’s and Colitis (PREdiCCt) study [98] and the Diet, microbiome and inflammatory bowel disease course (microIBDiet) study. The FARMM study, part of the CCFA’s Microbiome Initiative, is a controlled feeding experiment among healthy volunteers, with the objective of examining how different diets (the “Western” diet, exclusive enteral nutrition and a vegan diet for two weeks) influence the gut microbiota and faecal metabolomics. The GEM Project is recruiting healthy first-degree relatives of CD patients, and will assess genetic and environmental factors and the gut microbiome; participants will be followed up to assess the risk of developing CD and the factors associated with the disease. The PREdiCCT study will recruit IBD patients in remission, and will assess diet, lifestyle, genetics and microbiome; participants will be followed up for two years to study the factors associated with relapse. The microIBDiet study will recruit newly diagnosed IBD patients, and will assess diet and the microbiome; patients will be followed up for five years to study the factors associated with an impaired outcome (personal communication from the principal investigator). These projects promise to shed more light on the role of dietary and nutritional factors in IBD as a basis for further dietary intervention trials.

## 8. Conclusions

In summary, rapid technological bioscience development has opened up new horizons for an improved understanding of the role of gene–environment interactions in the onset and development of IBD. Targeted nutrition taking into account individual genetic make-up, epigenetics and microbiota composition may represent a novel platform for successful prevention and disease control. More work is required to evaluate the role of individual food compounds and complex nutritional interventions with potential to decrease inflammation, modulate immune-modulatory epigenetic traits and maintain intestinal microbial balance as a means of prevention and management of IBD.
